# Upcycling waste iron into high-performance Fe_3_Si-SiC-NbC in-situ nanocomposites enhances multiple properties during carbothermal reactions

**DOI:** 10.1038/s41598-026-53759-y

**Published:** 2026-06-02

**Authors:** Mohammed A. Taha, S. A. Gad, Rehab E. A. Ngida, Rasha A. Youness

**Affiliations:** 1 Solid State Physics Department, National Research Centre, El Buhouth St., Dokki, Giza, 12622 Egypt; 2 Pharos University in Alexandria, Canal Mahmoudiah Street, Smouha, Alexandria Egypt; 3 Refractories, Ceramics and Building Materials Department, National Research Centre, Dokki, Cairo, 12622 Egypt; 4 Spectroscopy Department, National Research Centre, El Buhouth St., Dokki, Giza, 12622 Egypt; 5 Centre for Converging Sciences and Emerging Technology (CoSET), Benha National University (BNU), Al Obour, 13518 Egypt

**Keywords:** Metal waste, Fe_3_Si intermetallic, Mechanical properties, Magnetic properties, Carbothermal reactions, Powder metallurgy, Engineering, Materials science

## Abstract

In this study, we are interested in reusing industrial waste to produce cost-effective Fe_3_Si intermetallic-based nanocomposites with excellent mechanical, thermal, and magnetic properties, fabricated through an in-situ carbothermal reaction during powder metallurgy sintering. Fe15Si5Nb (vol%) powder is milled with increasing proportions of activated carbon up to 8% using high-energy milling, then pressed into tablets and sintered in an inert gas. The microstructure and phase composition of the sintered sample were investigated with FESEM and XRD techniques. Moreover, physical, thermal, mechanical, and magnetic properties were studied. The results indicated that the particle size of the Fe_15_Si_5_Nb powder decreased with the addition of activated carbon during milling. After sintering, XRD results showed the formation of two phases: Fe₃Si and FeNb. Upon adding activated carbon, the FeNb phase dissolved, and ceramic phases, SiC and NbC, were formed. Furthermore, there was a marked improvement in both the thermal expansion coefficient (CTE) and mechanical properties, and also no breakdown of magnetic properties. The microhardness, strength, Young’s modulus, and CTE of the sample containing 8% activated carbon improved by approximately 69.81%, 33.95%, 21.77%, and 16.67%, respectively, compared to the base sample. Magnetization of the intermetallic base decreases from 28.12 to 26.20, 26.08, 23.65, and 21.98 emu/g, respectively, after incorporating 1%, 2%, 4%, and 8% activated carbon.

## Introduction

The iron (Fe) industries, as well as the construction, demolition industries, and metalworking shops, generate a substantial amount of iron waste. To date, the accumulation of these residues in the environment has not received the attention it deserves^1–4^. Recycling Fe is recognized as an environmental issue in industrial and civil building regions, contributing to sustainability by reducing production costs, energy consumption, and carbon dioxide emissions^5–8^. Recycling used metals into new materials is beneficial because the leading manufacturers of these materials require a large quantity of energy and raw resources. Recently, several researchers have developed hybrid nanocomposites using the powder metallurgy process. These materials utilize iron waste powder and exhibit improved mechanical, thermal, and tribological properties for industrial applications^4,9–11^. Considerable attention has been devoted to Fe–Si intermetallic compounds owing to their varied magnetic, electrical, mechanical, and catalytic capabilities, which stem from differing crystal structures and phase compositions. Fe–Si silicides of various compositions, including metastable Fe_2_Si, Fe_5_Si_3_, and α-FeSi_2_, as well as thermodynamically stable Fe_3_Si, FeSi, and β-FeSi_2_, have been synthesized and characterized^12–14^. The Fe–Si phase diagram^13^ shows that FeSi has a relatively narrow homogeneity range, while Fe_3_Si has a broader stoichiometric range (10 to 29.8% Si). In the range of 25 to 50 at% Si, both phases can coexist. At a temperature above 937 °C, α-phase FeSi_2_ forms in the range of 70–73.5 at% Si. In contrast, β-FeSi_2_ is a line compound at 66.7 at% Si and remains stable at ambient temperature^14^. The mechanical behavior and microstructural development of Fe–Si alloys are greatly impacted by the addition of niobium (Nb). Strong alloying elements like Nb easily combine with Fe to create stable intermetallic compounds like FeNb^15–17^. Through dispersion strengthening and grain boundary pinning effects, the FeNb phase, renowned for its high melting point, chemical stability, and exceptional hardness, enhances the alloy’s strength and resistance to wear. Furthermore, at higher temperatures, the production of FeNb results in decreased diffusion rates and improved thermal stability. Consequently, Nb addition is a valuable method for producing Fe–Si–based alloys with exceptional mechanical and thermal properties, suitable for high-performance structural and electronic applications^15,18^.

High strength, remarkable thermal stability, and outstanding oxidation resistance are just a few of the intriguing properties of intermetallic compounds. Despite these advantages, their properties are still insufficient for many advanced technical applications. These include resistance heating elements, furnace components, transformer cores, inductors, electromagnetic devices, valve system parts, exhaust pipes, and turbine heat exchangers, which require higher wear resistance, better thermal performance, and improved mechanical reliability. To satisfy the requirements of contemporary applications, which often call for modifying their microstructure and adding extra reinforcing phases to obtain better overall performance and improve the required properties. Moreover, it can be used effectively in different mid-frequency magnetic components. Applications that require a balance between mechanical properties, thermal stability, and acceptable magnetic performance include chokes, small and micro motors, inductors, magnetic sensors, and functional magnetic components^8,19–21^.

Activated carbon, characterized by its high surface area and chemical reactivity, is a key precursor for in-situ carbide formation. Its incorporation into metal powder mixtures enables effective reactions during high-temperature sintering, producing hard ceramic phases such as SiC and NbC. These carbothermal reactions generate reinforcing ceramics within the matrix, improving phase distribution and interfacial bonding, thereby enhancing the composite’s overall properties^22^. Powder metallurgy, through controlled chemical composition and microstructural development, provides an efficient method for producing advanced metallic and ceramic-reinforced composites^23–26^. Reactive sintering, a notable powder metallurgy technique, effectively densifies powders and fosters in situ phase creation. This process leverages the proximity of powder particles to enhance bonding and lead to beneficial diffusion-driven changes, resulting in complex multi-phase composites with optimized mechanical, thermal, and functional properties^27–29^.

The current study is expressly differentiated from prior research on Fe–Si-based composites. This work examines the in-situ synthesis of a ternary Fe_3_Si–SiC–NbC nanocomposite via a controlled reaction route, contrasting with previous research that mainly concentrated on binary Fe–Si systems or the ex-situ incorporation of reinforcing phases. A notable feature of this research is the use of recycled scrap iron as the principal raw material, which contributes to its sustainability and industrial significance. The key novelty lies in (i) the concurrent development of dual carbide phases (SiC and NbC) within a Fe₃Si matrix, (ii) the influence of carbon content on phase evolution and microstructural refinement, and (iii) the attainment of a highly refined, diffusion-controlled microstructure that improves densification and phase distribution. These elements combined distinguish our study from other documented Fe–Si-based composites and provide novel insights into in-situ nanocomposite production methods. Furthermore, the prepared nanocomposite samples have excellent mechanical properties and thermal stability, while at the same time not losing their magnetic properties, which allows their use in the various applications mentioned above.

## Materials and experimental setup

### Sample preparation

**Fig. 1:**
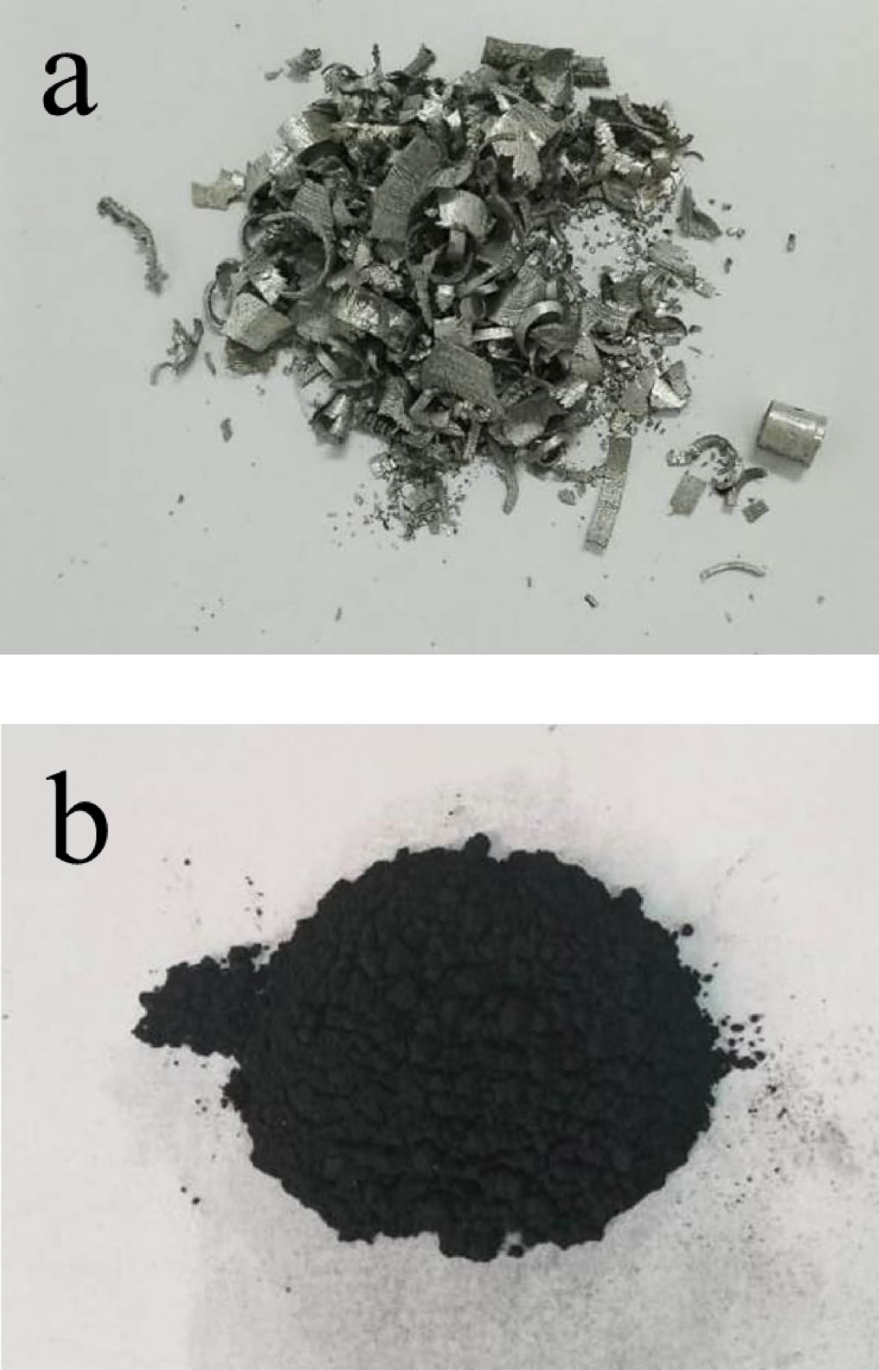
Photos of Fe waste (**a**) generated in lathe workshops and (**b**) after milling 20 h.

The Fe scrap from the lathe workshops, which is composted in Table 1, was broken down into small pieces, several millimeters in size, using a ball mill for 3 h with a ball-to-powder ratio (BPR) equal to 10:1 and a speed of 400 rpm (Fig. 1). It was considered a raw material for preparation. Furthermore, Si and Nb were used as matrix elements, while activated carbon was used as reinforcement. The base matrix of this work consisted of FeSi15Nb5 (vol%) alloys, which were mixed for 5 h with a BPR of 5:1 and a speed of 150 rpm. Secondly, varied contents of the activated carbon were added to the FeSi15Nb5 alloy matrix. The batch compositions designed for milled powders, with their abbreviations, are listed in Table 2. Each sample powder was milled for 20 h at 400 rpm with a ratio of balls to powder = 20:1, having in mind that the milling process was done in a cycle of 2 h and paused for 1 h. The powder was compacted with a hydraulic machine with a pressure of 400 bar and then sintered in an argon atmosphere for 2 h at 1200 °C with a heating rate of 5 °C/ min.

**Table 1:** Composition of Fe waste powders (wt%).

Element	Fe	Mn	Al	*P*	C	other
wt%	99.75	0.06	0.05	0.04	0.04	0.06

**Table 2:** Batch design of the prepared samples (vol%).

Sample name	Fe_15_Si_5_Nb (vol%)	Activated carbon
Sample 1	100	0
Sample 2	99	1
Sample 3	98	2
Sample 4	96	4
Sample 5	92	8

### Particle size and phase composition

The particle size distribution curves of the raw materials and all mill samples were measured using a diffraction particle size analyzer. The phase composition of raw materials (Fe, Si, Nb, and activated carbon) and each milled and sintered sample was determined using the X-ray diffraction (XRD; Philips PW 1373 X-ray powder diffractometer) technique equipped with a Ni-filter and Cu-Kα radiation (λ = 1.5406 Å). The scanning was performed over a 2θ range of 20° to 90° and a scanning rate of approximately 0.5°/min. The operating conditions were set at 40 kV and 30 mA.

### Physical properties

The bulk density (BD), relative density (RD), and total porosity (TP) of each sintered sample were assessed by the Archimedes technique and calculated using the formulas provided below^30^:

1 $$\:\mathrm{B}\mathrm{D}=\:\frac{{\mathrm{W}}_{\mathrm{d}}-{\mathrm{W}}_{\mathrm{s}}}{{\mathrm{W}}_{\mathrm{s}}-{\mathrm{W}}_{\mathrm{i}}}\:\times\:{{\uprho\:}}_{\mathrm{l}}$$

2 $$\:\mathrm{R}\mathrm{D}=\:\frac{\mathrm{B}\mathrm{D}}{\mathrm{R}\mathrm{D}}\times\:100$$

3 $$\:\mathrm{T}\mathrm{P}=\left(1-\:\frac{\mathrm{B}\mathrm{D}}{\mathrm{T}\mathrm{D}}\right)\times\:100$$

Where W_d_, W_s_, and W_i_ are the weights of dry, saturated, and immersed samples, respectively, and ρ_l_ is the density of the liquid. TD is the theoretical density, which is calculated using a mature rule.

### Microstructure of sintered samples

The microstructure of the sintered samples was examined using field-emission scanning electron microscopy with energy-dispersive X-ray analysis (FESEM-EDS; Quanta FEG250, operated at an accelerating voltage of 30 kV). In particular, samples were washed with ethyl alcohol, left to dry, and then coated with a thin layer of gold to enhance their brightness before being analyzed with a FESEM.

### Thermal properties

Thermal expansion measurements of all sintered samples were analyzed over the temperature range of 30 to 800 °C at a rate of 5 °C/min. The coefficient of thermal expansion (CTE) value is calculated based on the linear length changes using the equation^31^:

4 $$\:\mathrm{C}\mathrm{T}\mathrm{E}=\frac{1}{\mathrm{L}}\:\frac{\mathrm{d}\mathrm{l}}{\mathrm{d}\mathrm{T}}$$

The variables L, dl, and dT represent the original length, the change in length, and the temperature change, respectively.

### Mechanical properties

As mentioned in our recent work^32^, microhardness (HV) of the sintered samples was measured by a Vickers tester (model: Shimadzu Corporation hardness tester) according to ASTM: B933-09 with an applied load (P) of 1.9 N for 10 s:

5 $$\:\mathrm{H}\mathrm{V}=1.854\:\times\:\frac{\mathrm{P}}{{\mathrm{d}}^{2}}$$

Where d is the diagonal of indentation.

The velocities of ultrasonic waves, i.e., longitudinal and shear waves propagating in the specimens, were obtained using the pulse-echo technique (MATEC Model MBS8000 DSP) at room temperature, as presented in the data^33,34^. As mentioned in our previous works^35,36^, the group of elastic moduli, including longitudinal modulus, Young’s modulus, bulk modulus, shear modulus, and Poisson’s ratio, was calculated. The mechanical tester (Instron, Darmstadt, Germany) was used to evaluate the sintered samples’ compressive strength (σ). One millimeter per minute was the continuous pace at which the compression tests were conducted, as measured using the following equation^37^:

6 $$\:{\upsigma\:}=\frac{\mathrm{F}}{\mathrm{A}}$$

F represents the load at fracture, and A represents the disc surface area.

### Magnetic properties

A vibrating sample magnetometer (VSM; Lake Shore-7410-USA) was used to investigate the magnetic characteristics of all synthesized samples. A vibrating sample magnetometer (VSM, Lake Shore 7410, USA) was used to evaluate the magnetic properties of each sample. Magnetic hysteresis (M-H) loops were recorded for each sample under study. Fundamental magnetic parameters, such as saturation magnetization (Ms), residual magnetization (Mr), magnetic coercivity (Hc), and the squared ratio (Mr/Ms), were extracted from the recorded M-H curves. All measurements were performed at room temperature (approximately 300 K), with the applied magnetic field varying within ± 20 kG. 50 mg from Fe, Si, Nb, and activated carbon powders fixed in a non-magnetic holder to ensure measurement stability.

## Results and discussion

### Particle size distribution

**Fig. 2:**
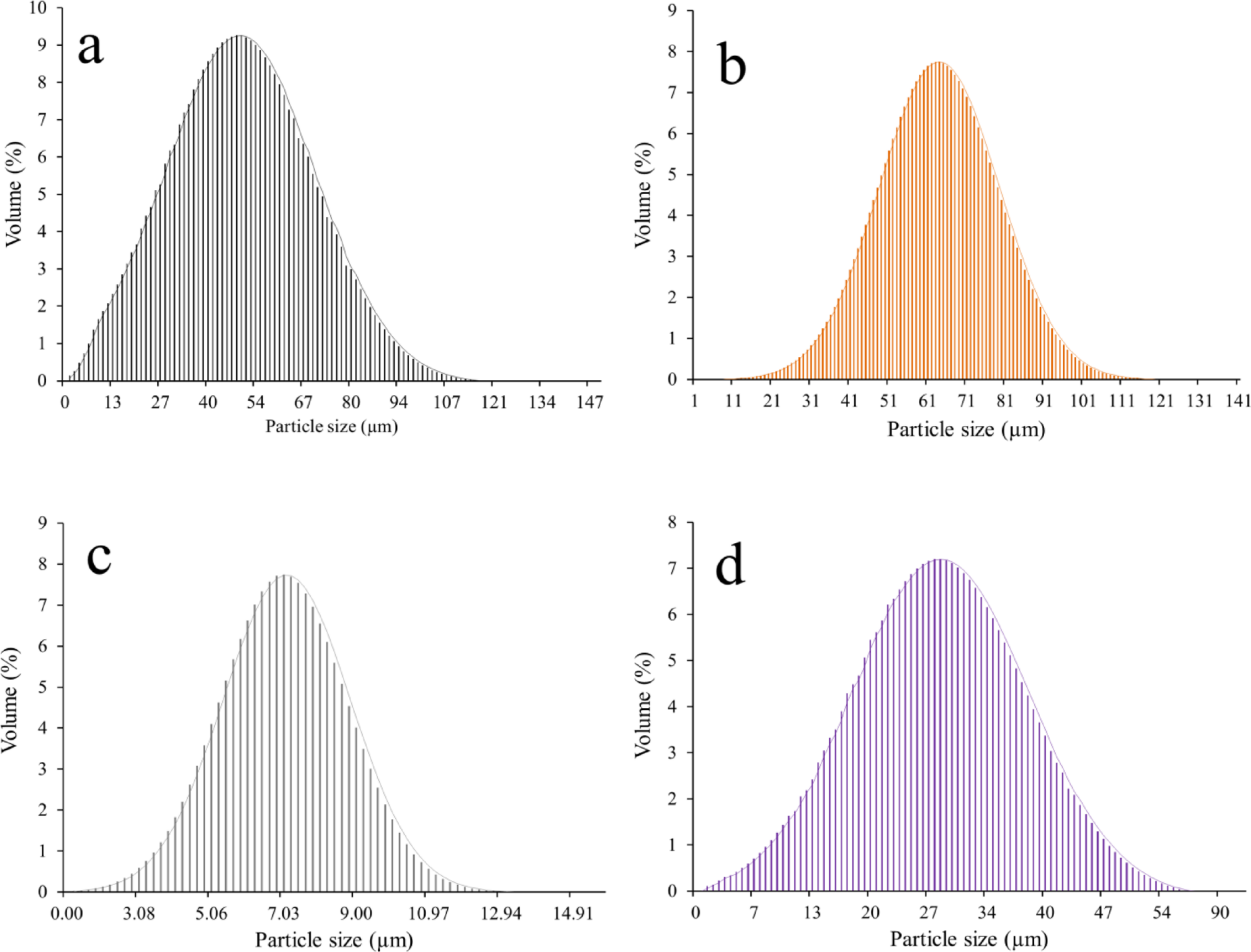
Particle size distribution of raw materials: (**a**) Fe, (**b**) Si, (**c**) Nb, and (**d**) activated carbon.

**Fig. 3:**
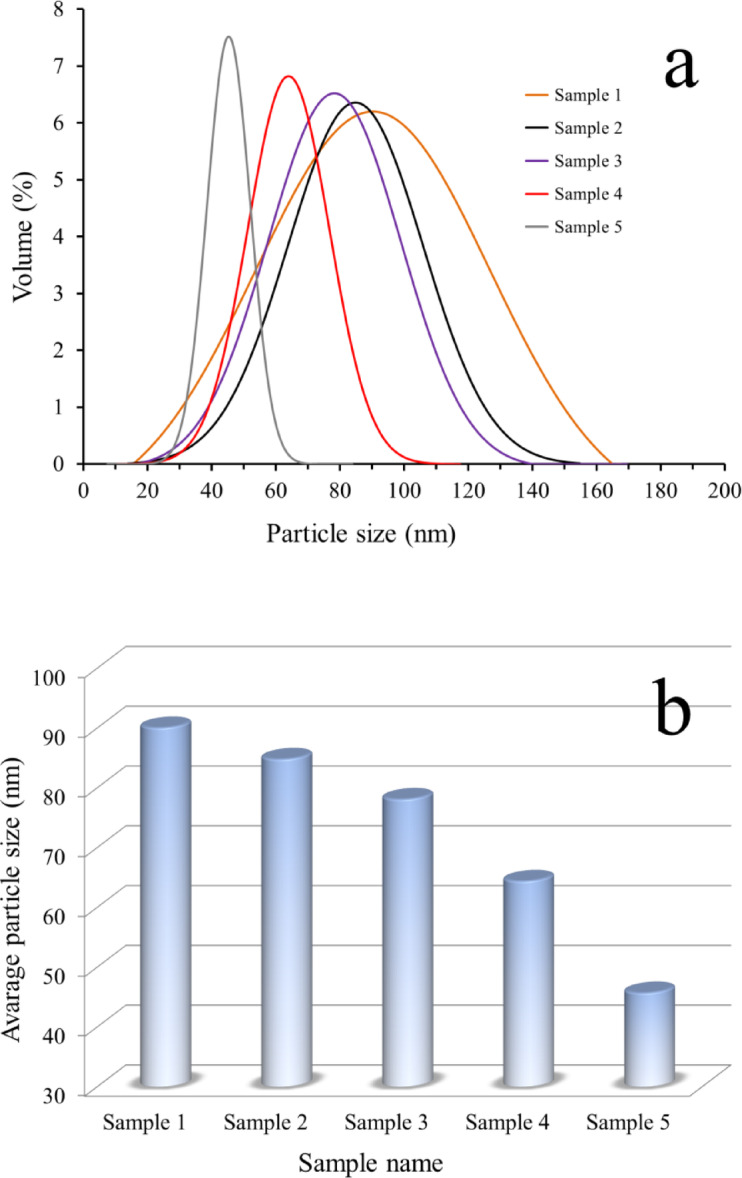
(**a**) Particle size distribution and (**b**) average particle size of all prepared samples after milling for 20 h.

Figure 2 illustrates the particle size distribution of Fe, Si, Nb, and activated carbon raw powders. The average particle size of the previous powders is 50.13, 63.22, 7.22, and 28.04 μm, respectively. Figure 3a illustrates the particle size distribution for all samples after 20 h of milling. The particle size distribution curves demonstrate that adding more activated carbon significantly refines the Fe–Si–Nb alloy powder. Because of the intense cold welding and the agglomeration of the metallic particles during milling, Sample 1, which is without activated carbon, has the greatest particle size and the widest dispersion. The distributions become narrower, and the particle size continues to decrease as activated carbon is progressively introduced (Samples 2–5). Activated carbon’s very hard and de-agglomerating properties, which lessen particle-particle stickiness and inhibit cold welding, are primarily responsible for this behavior. Such behavior enables the milling balls to fracture the powders more successfully. Consequently, smaller, more consistent particle sizes result from increased carbon concentrations. The mean particle size values of the Samples from 1 to 6 were 90.05, 84.78, 78.08, 64.39, and 45.70 nm, respectively, as shown in Fig. 3b.

### Phase analysis

**Fig. 4:**
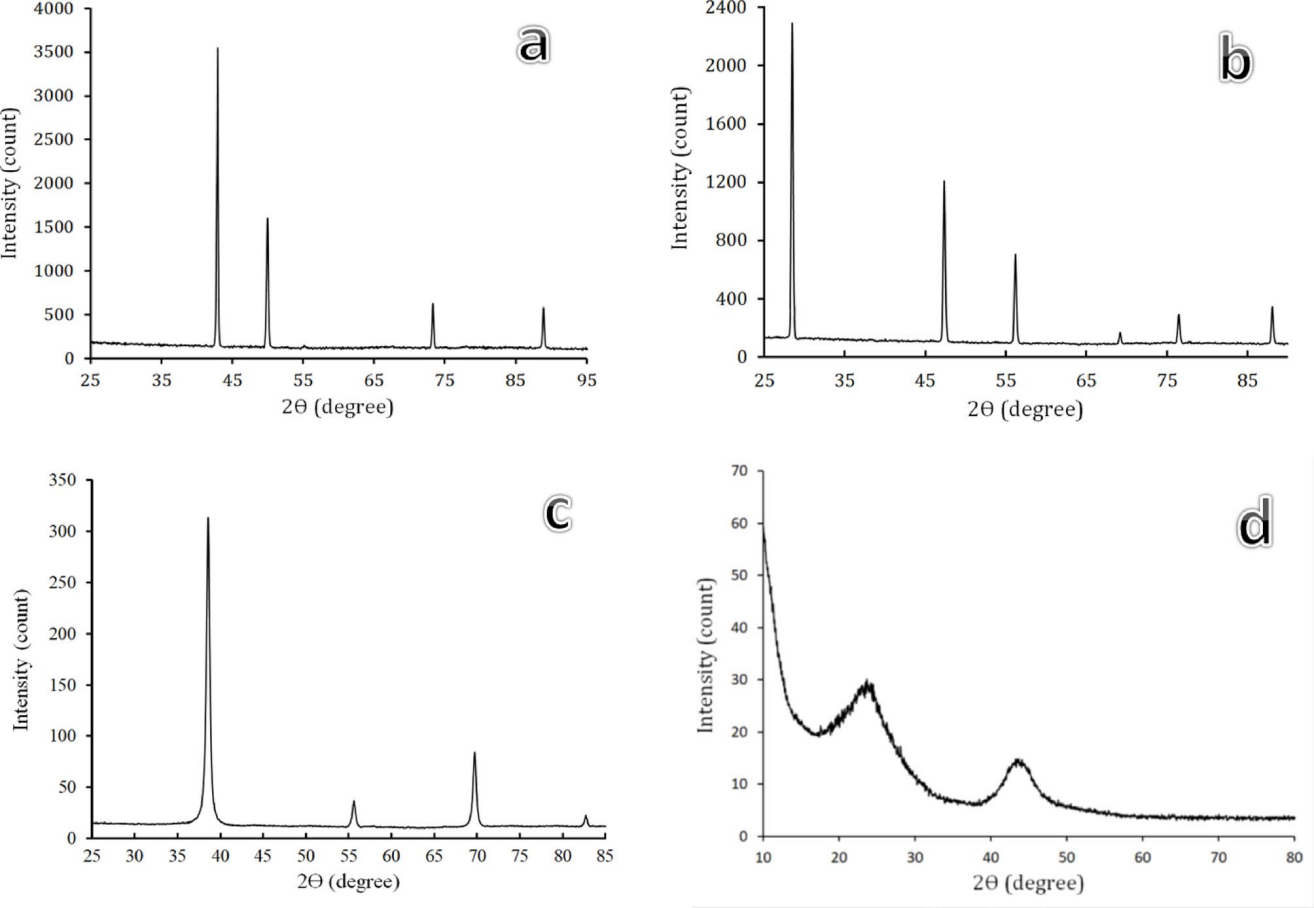
XRD patterns of raw materials (**a**) Fe, (**b**) Si, (**c**) Nb, and (**d**) activated carbon.

Figure 4a-d shows the XRD patterns of the starting materials, including Fe, Si, Nb, and activated carbon powders. For Fe, Si, and Nb, as shown in Figs. 4(a-c), the patterns reveal the presence of characteristic peaks according to ICCD file cards 89-4185, 89-5012, and 89-5008, respectively. According to the XRD cards, the Fe, Si, and Nb powders exhibit a cubic crystal structure. The peaks of the Fe phase are clearly identified at 2θ = 42.90°, 49.95°, 38.42°, 40.17°, 73.33°, and 88.89°, and Si are 28.4°, 47.30°, 56.12°, 69.13°, 76.38°, and 88.03°, and Nb are 38.61°, 55.76°, 55.76°, 69.87°, and 82.79°, respectively. Conversely, in Fig. 4d, the absence of discernible diffraction peaks indicative of lattice periodicity in activated carbon implies an amorphous structure.

**Fig. 5:**
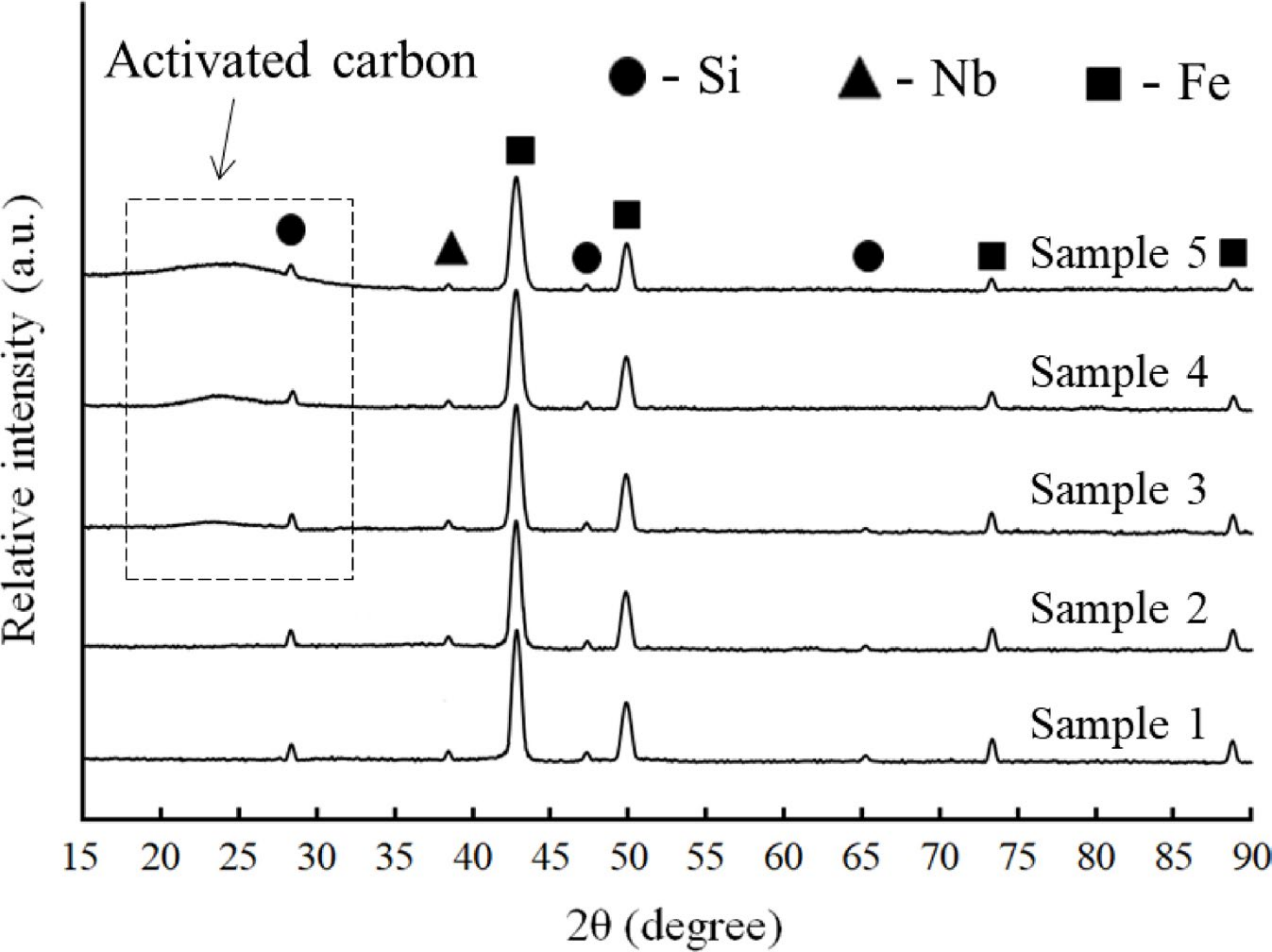
XRD patterns of the Fe15Si5Nb alloy and its nanocomposite powders with varying activated carbon contents after 20 h of milling.

After 20 h of mechanical milling, the XRD patterns in Fig. 5 depict the phase evolution of Fe—Si—Nb alloy with the addition of various activated carbon amounts (0, 1, 2, 4, and 8 vol%). The diffraction peaks for Sample 1 belong to Fe-based phases together with Si and Nb. Because activated carbon is amorphous, it causes a wide hump to emerge in the range of around 2θ ꞊ 20°–30°. As the activated carbon concentration rises, this hump gets more pronounced, indicating that non-crystalline carbon is present in the system. Concurrently, when the activated carbon concentration rises, the diffraction peaks widen and become somewhat less intense. This behavior is mostly explained by the effects of 20 h of high-energy ball milling, which causes lattice strain and grain size refinement. Furthermore, the absence of recognizable carbide ceramic peaks at this point indicates that the system is still mechanically alloyed and that no carbothermal reaction has taken place during milling.

**Fig. 6:**
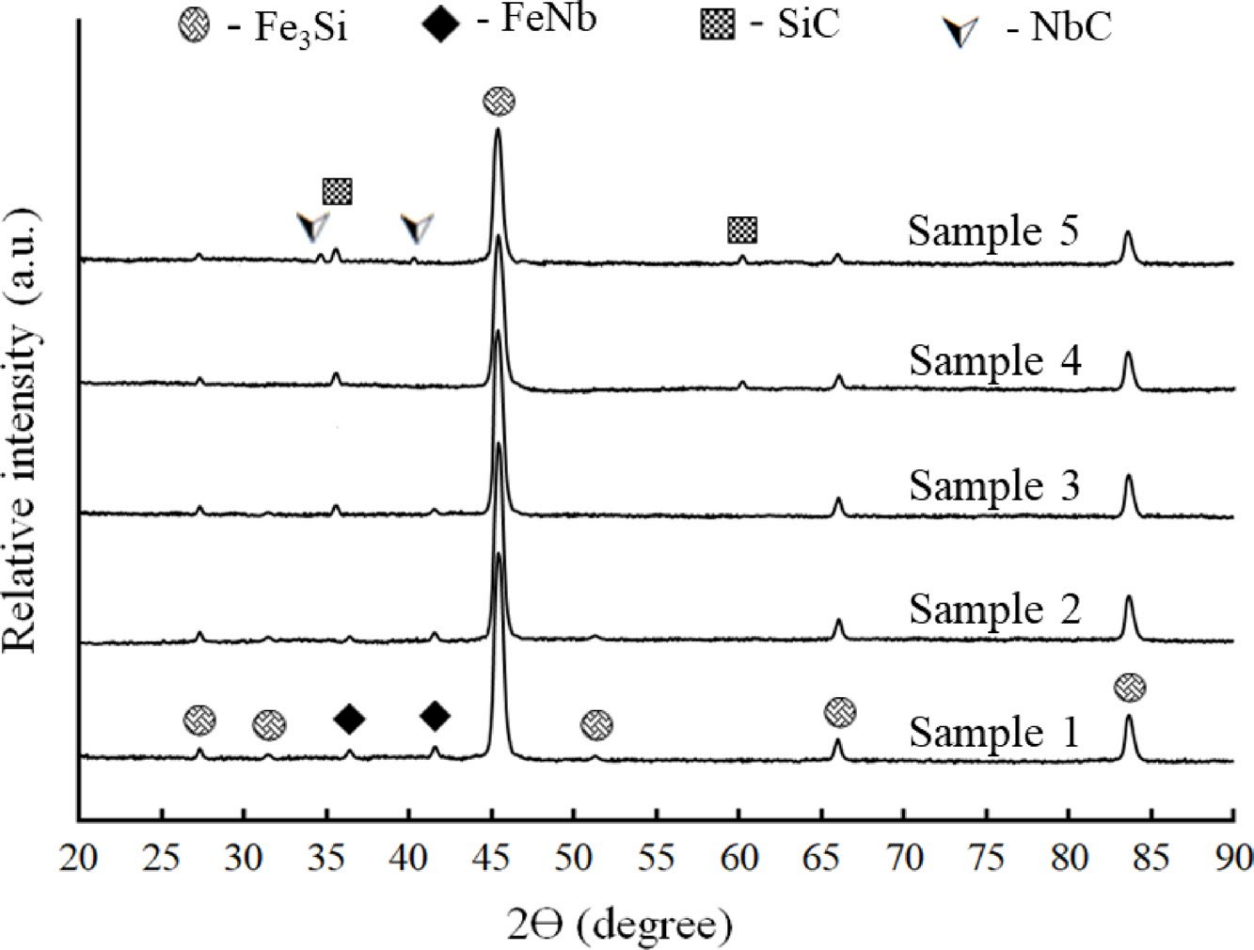
XRD patterns of all samples after sintering at 1200 °C for two hours in an argon atmosphere.

Figure 6 shows the XRD of the Fe-Si-Nb alloy sample with different contents of activated carbon up to 8 vol% after sintering at 1200 °C for 2 h in an argon atmosphere. The XRD patterns of the examined samples clearly demonstrate a gradual phase development corresponding to the increasing activated carbon amount. In the carbon-free sample (Sample 1), the microstructure mainly consists of Fe₃Si phase with minor FeNb intermetallics (ICCD file cards 65-3005 and 65-3201, respectively), confirming the stability of the base alloy system without activated carbon. A semi-quantitative assessment of the relative intensities of the principal diffraction peaks was performed to monitor phase progression (Table 3). The findings demonstrate that Fe_3_Si persists as the predominant phase throughout all samples, although with a progressive decline in relative intensity as activated carbon content increases. This indicates the partial consumption of Fe_3_Si during activated carbide production instead of a full phase change. The introduction of activated carbon (Samples 2–5) results in the progressive emergence of additional diffraction peaks corresponding to SiC and NbC, as per standard cards 88-2514 and 85-5514, respectively, with an increase in intensity, thereby confirming the in-situ formation of carbides during sintering at 1200 °C. The observed pattern indicates an ongoing interaction between carbon and the alloying elements, with Si and Nb serving as reactive agents for carbide production. The thermodynamic favorability of SiC production arises from the strong affinity between silicon and carbon, facilitating the in-situ reaction when free or partly freed silicon is sourced via the breakup or redistribution of Fe–Si intermetallics (e.g., Fe_3_Si). Conversely, NbC formation is mostly influenced by the strong chemical affinity between niobium and carbon; yet, its production is more kinetically constrained because of the reduced diffusivity of Nb relative to Si. Consequently, SiC tends to nucleate more promptly and easily, while NbC production requires extended diffusion durations and localized carbon enrichment. The disparity in thermodynamic and kinetic behavior elucidates the observed phase development and the corresponding intensities of the carbide phases as carbon concentration increases. Conversely, whereas silicon has greater mobility that kinetically promotes SiC production, the overall process results in the simultaneous synthesis of both carbide phases, albeit with restricted volume fractions even at elevated carbon concentrations. The combined qualitative and semi-quantitative research further substantiates that elevated activated carbon content facilitates the in-situ synthesis of SiC and NbC, detracting from the Fe_3_Si matrix. The development of phases is dictated by a balance between thermodynamic driving forces and kinetic constraints related to elemental diffusion during sintering.

**Table 3:** Comparative semi-quantitative estimation of phase fractions derived from XRD peak intensity analysis, illustrating the evolution of Fe_3_Si, SiC, and NbC phases with increasing activated carbon content.

Sample name	Fe_3_Si (%)	SiC (%)	NbC (%)	FeNb (%)
Sample 1	~ 95–97	0	0	~ 3–5
Sample 2	~ 93–95	~ 2–3	~ 1–2	trace
Sample 3	~ 92–94	~ 3–4	~ 1–2	0
Sample 4	~ 90–93	~ 4–5	~ 2–3	0
Sample 5	~ 88–92	~ 5–6	~ 2–3	0

### FESEM analysis

**Fig. 7:**
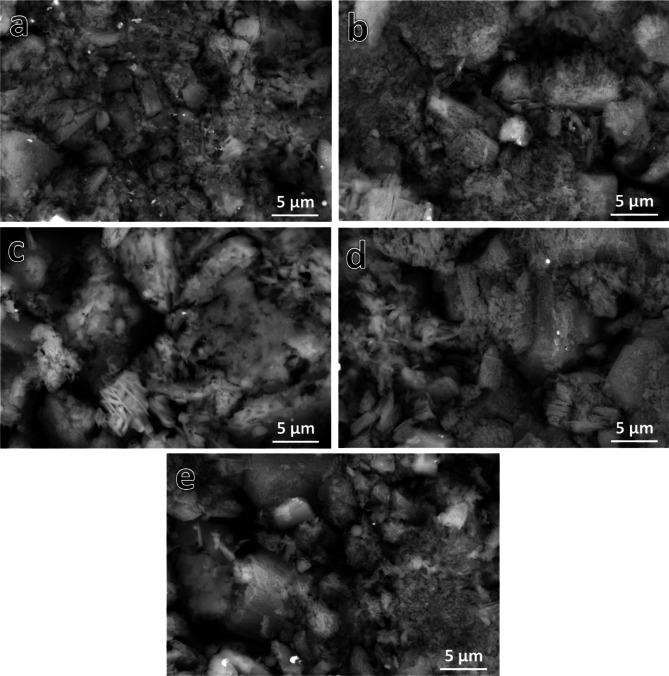
FESEM images of (**a**) sample 1, (**b**) sample 2, (**c**) sample 3, (**d**) sample 4, and (**e**) sample 5 after sintering.

**Fig. 8:**
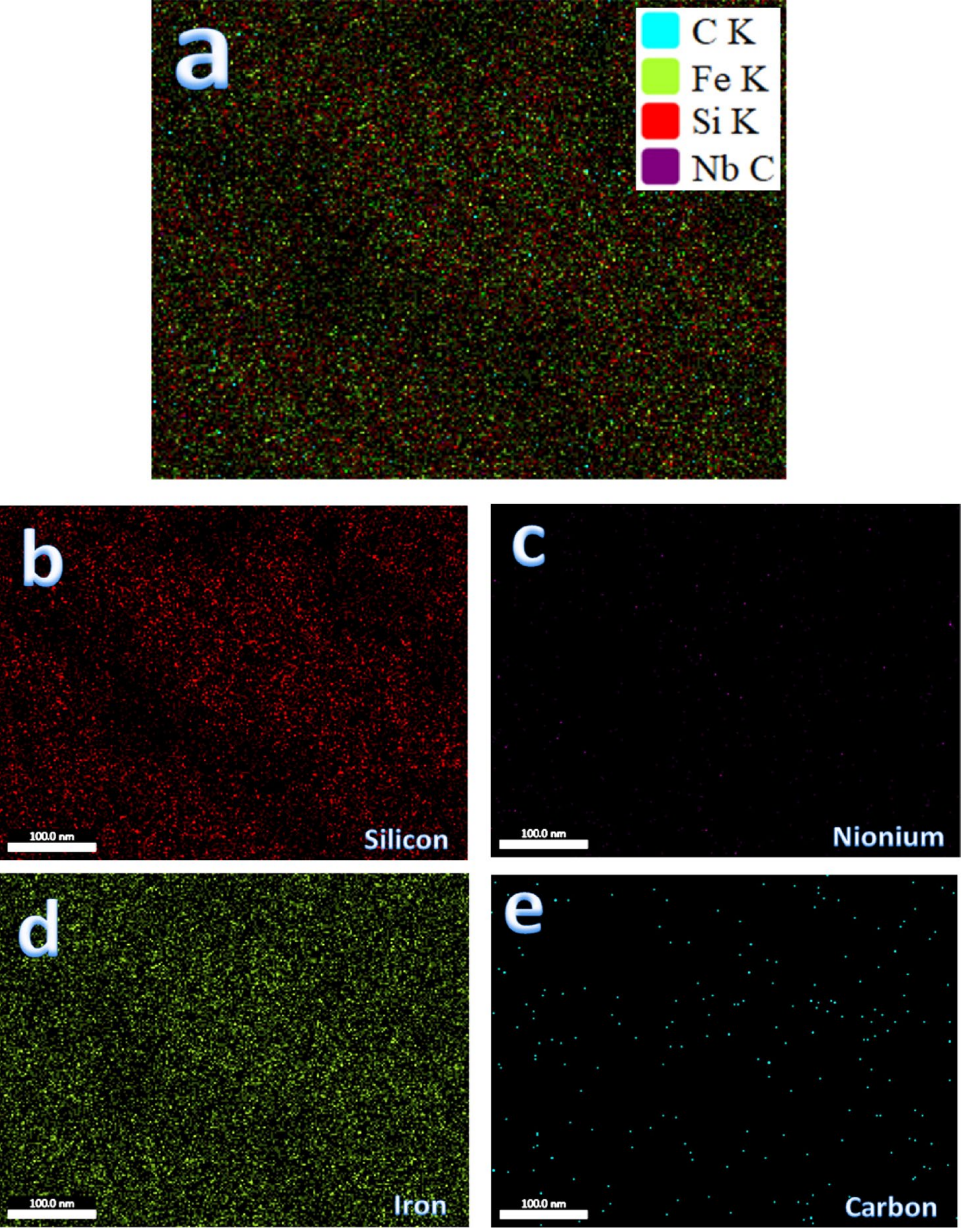
(**a**) EDX mapping of all constituents of sample 5, and elemental mapping of the constituents forming sample, i.e., (**b**) Fe, (**c**) Si, (**d**) Nb, and (**e**) activated carbon.

Figure 7(a-e) represents SEM images of sintered Samples 1–5, respectively. The selection of a sintering temperature of 1200 °C facilitates the diffusion process during sintering, enhances densification behavior, and approaches optimal density values. The intermetallic alloy (Sample 1) demonstrated adequate densification, as indicated by notable particle growth and a limited presence of pores (Fig. 7a). In the nanocomposite samples depicted in Fig. 7b and e, reinforcement (SiC and/or NbC) particles are observed at the grain boundaries of the intermetallic alloy matrix. The reinforcement particles were uniformly distributed within the intermetallic matrix due to effective mixing at the optimal milling time, which promoted the formation of SiC and NbC reinforcements and improved component wettability. The effective distribution of reinforcements within the intermetallic base is essential for enhancing the desired characteristics of the nanocomposite, including its thermal, mechanical, and magnetic properties. Increasing the ceramic particle concentration may lead to a reduction in particle grain size post-milling, potentially decreasing the extent of agglomerated regions remaining after sintering. The quantity of hybrid reinforcement particles in the samples was found to affect the porosity of the examined nanocomposite samples. Figure 8 displays the elemental mapping of Sample 5. Figure 8a shows the homogeneous distribution of all the particles composing Sample 5. The distribution of each element (Fe, Si, Nb, and C), and it is clear that the distribution is homogeneous, as shown in Fig. 8b and e.

### Physical properties

**Fig. 9:**
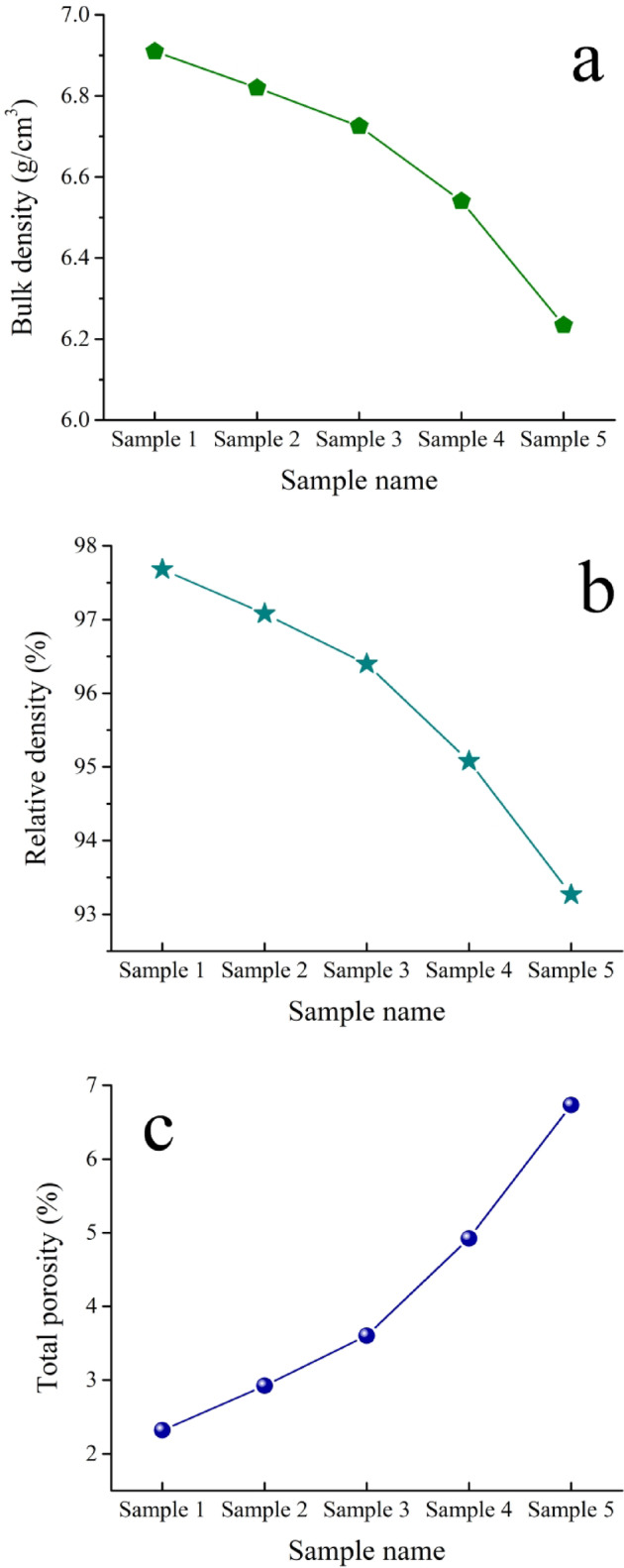
(**a**) Bulk density, (**b**) relative density, and (**c**) total porosity of all samples sintered.

Figure 9 illustrates the effect of various volume percentages of activated carbon on BD, TP, and RD of the intermetallic alloy-based nanocomposites sintered for 1 h at 1200 °C in an argon atmosphere. The theoretical densities of Samples 1, 2, 3, 4, and 5 are 7.074, 7.025, 6.977, 6.879, and 6.684 g/cm^3^, respectively. The results indicate that BD and TP for all composite samples decrease after the addition of activated carbon, while total porosity increases. The RD of the previous samples are 97.982%, 97.078%, 96.398%, 95.077%, and 93.268% while TB for the same samples are 2.318, 2.922, 3.602, 4.923, and 6.732 g/cm^3^, respectively. The decrease in BD and RD and the increase in TP with higher activated carbon content are attributed to the formation of ceramic phases (SiC and NbC) during sintering, weakening intermetallic phase bonding, restricting grain growth, and promoting closed-pore formation. The significant disparity between the melting temperatures of the intermetallic alloy matrix and the ceramic formed components results in less particle rearrangement during sintering. The findings presented here are in agreement with those of Refs^38–40^. For example, Wang et al.^41^ investigated the impact of SiC and TiC in situ formation on the relative density of iron matrix composites. They found that an increase in SiC and TiC formation resulted in a reduction in the composite’s relative density. Issa et al.^42^ reported that the relative density of Fe-Cu-based composites decreases with the addition of NbC nanoparticle reinforcements. Huang et al.^43^ studied the effects of the TiC content on the densification of TiC/Fe composites. The result indicated that the densification (relative density) of prepared composites decreased gradually with an increase in the TiC reinforcements.

### Thermal expansion

**Fig. 10:**
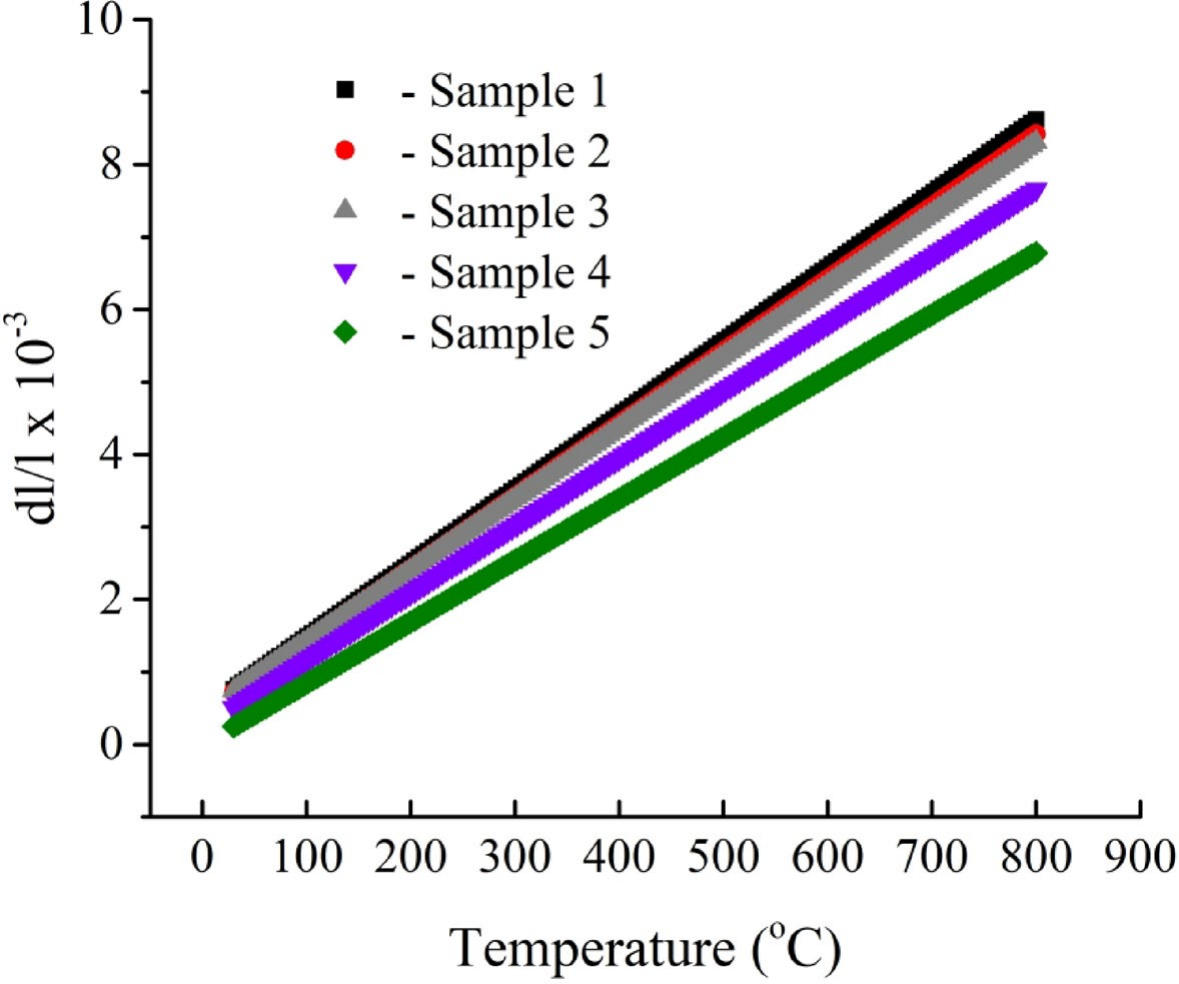
Relative thermal expansion for all sintered samples.

**Fig. 11:**
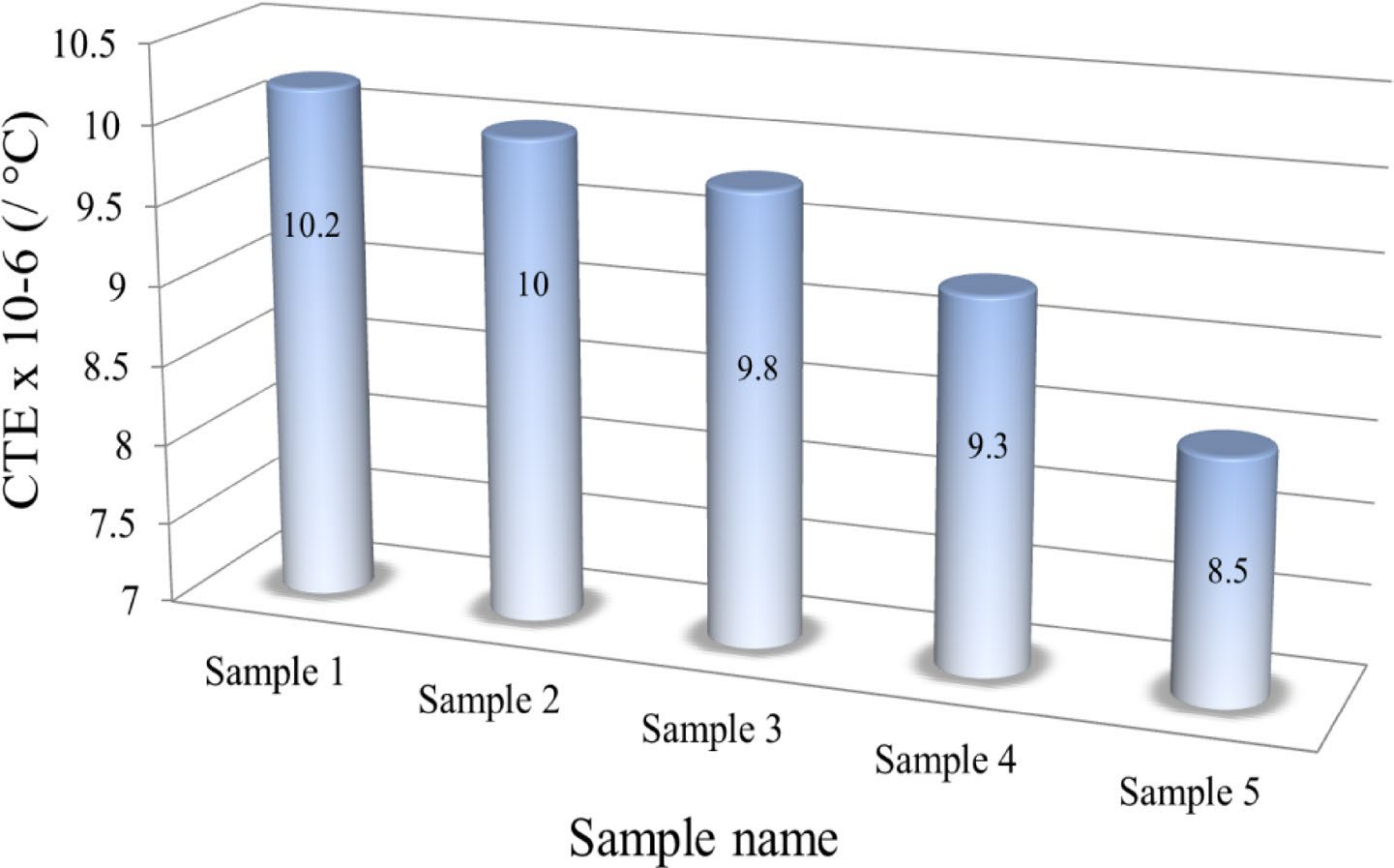
CTE value for all sintered samples.

The values of the coefficient of thermal expansion indicate the extent of mismatch strains introduced into the matrix, resulting either from the addition of reinforcements with differing properties or from microstructural changes^44,45^. Each phase possesses distinct physical properties, shapes, and volumes, which contribute to the residual stresses at the boundaries following thermal treatment. Residual stresses at the boundaries can be transmitted without the expansion of the intermetallic alloy or debonding of the boundaries^46,47^. Figure 10 illustrates the relative expansion (dl/l) as a function of temperature of all sintered nanocomposite samples. The curves found for the dl/l value increase consistently as the temperature continued to rise, and the slope of the line decreased with increased carbide ceramic reinforcements. Figure 11 illustrates the change in CTE value with increasing carbide ceramics formed in the samples, calculated from the previous figure. As can be observed, the CTE value of the intermetallic alloy matrix decreased with increasing reinforcement. The value of CTE of samples 2 to 5 is 10 × 10^− 6^, 9.8 × 10^− 6^, 9.3 × 10^− 6^, and 8.5 × 10^− 6^ /°C, which is a decrease of about 1.96%, 3.92%, 8.82%, and 16.68%, respectively, compared to Sample 1 (10.2 × 10^− 6^ /°C). The observed decrease in CTE value could be attributed to the fact that carbide reinforcements provide a drag force on the grain boundary motion that is more effective. In addition, reinforcements imposed a greater compressive strain on the grain boundaries during the expansion of intermetallic alloy-based nanocomposites as a result of the very low CTE that they had. The impact of this force that occurs throughout the expansion process is to restrict this particular characteristic of the intermetallic alloy-based. As a consequence, the composites’ CTE decreased^10,45^. Furthermore, the reduction in CTEs of metals and their alloy after the addition of carbide ceramic in this study was consistent with findings reported in the literature^9,48,49^.

### Mechanical properties

**Fig. 12:**
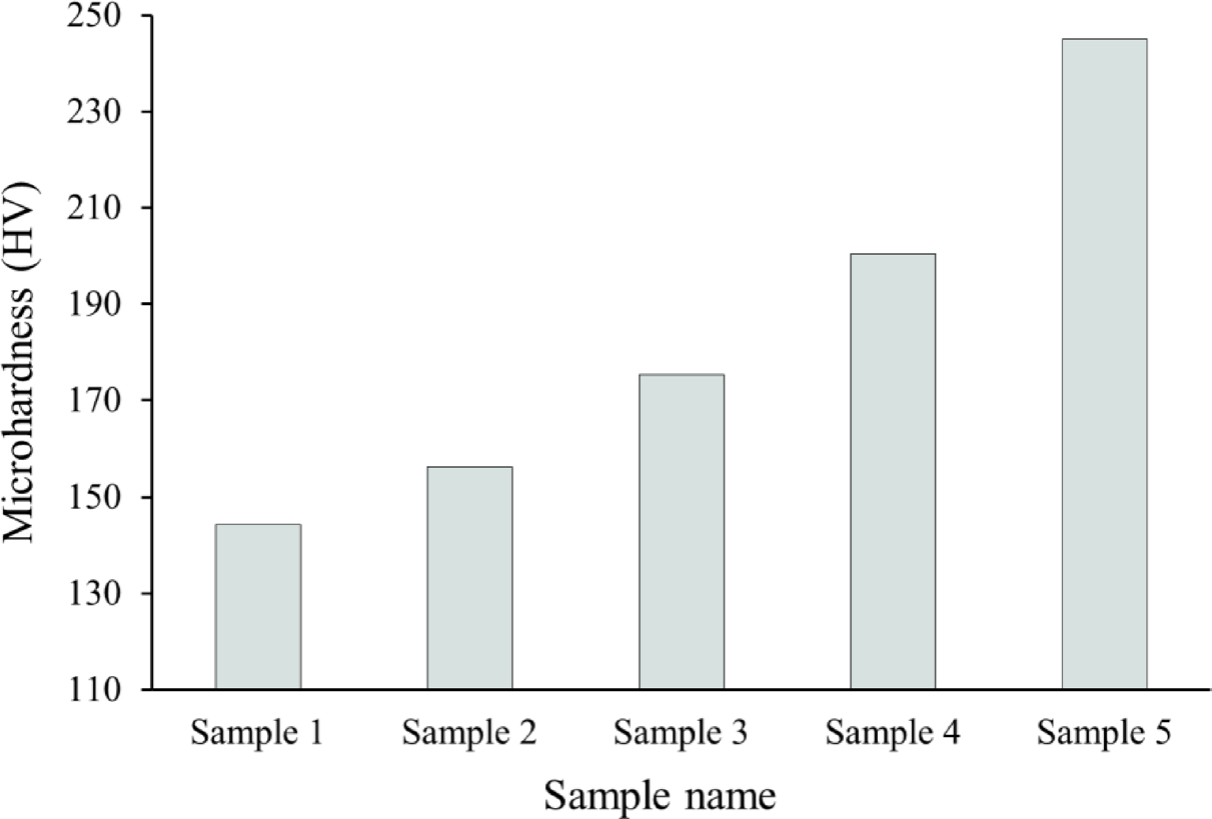
Effect of mono- and hybrid in-situ reinforcement on microhardness of all samples.

**Fig. 13:**
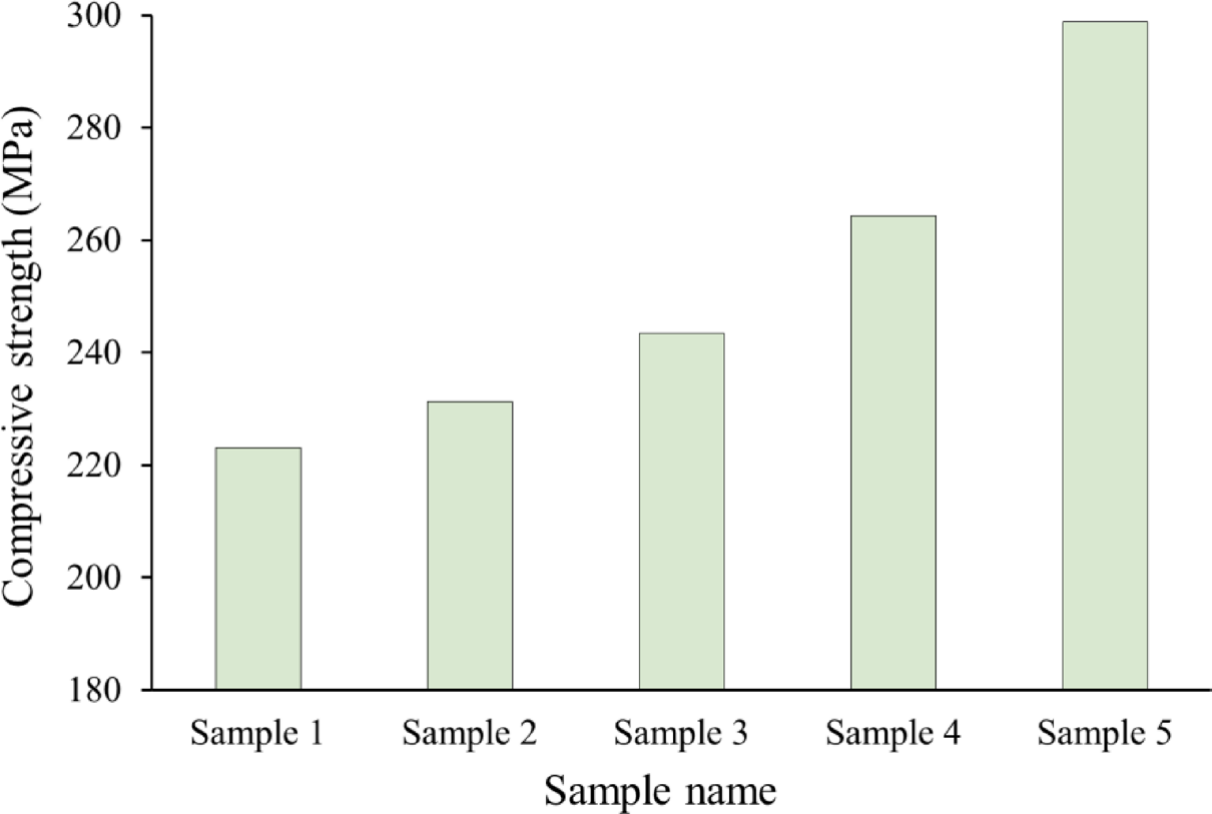
Effect of mono- and hybrid in-situ reinforcement on compressive strength of all samples.

**Fig. 14:**
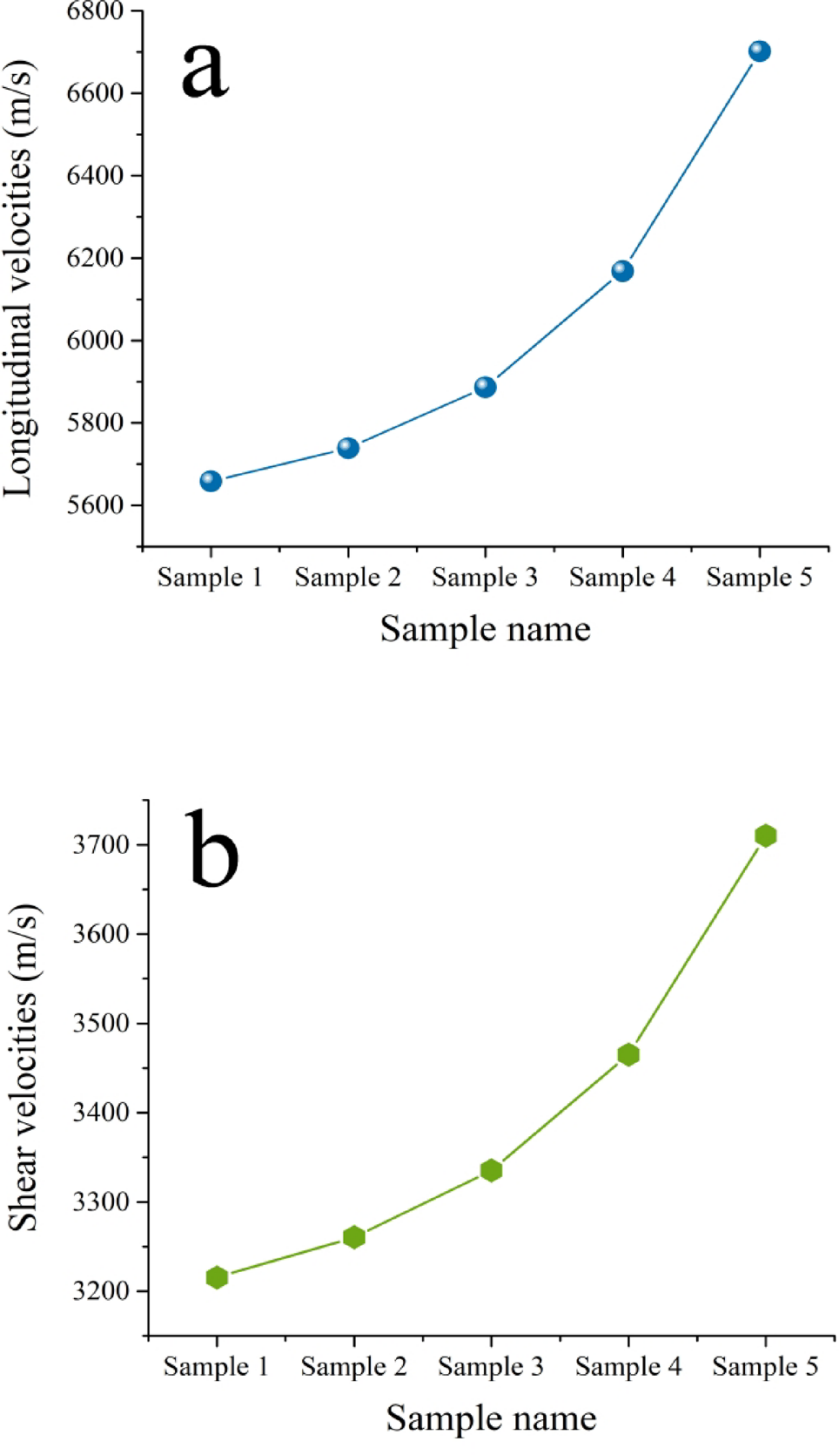
(**a**) Longitudinal velocity and (**b**) shear velocity of all sintered samples.

**Fig. 15:**
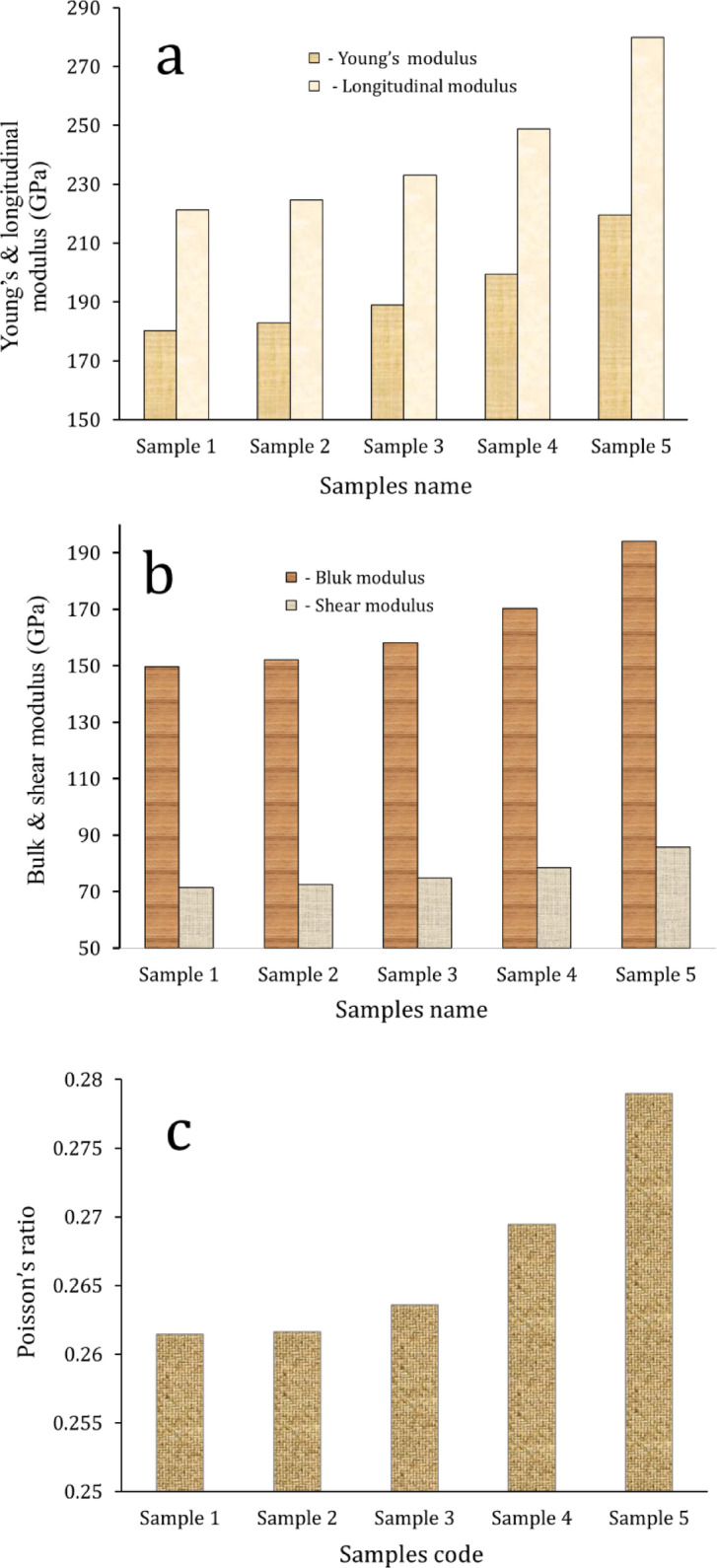
(**a**) Longitudinal and Young’s, (**b**) bulk and shear, and (**c**) passion ratio of all sintered .samples.

The introduction of ceramic reinforcement particles, such as SiC and NbC, into a metal and its alloys matrix primarily serves the critical function of improving the material’s mechanical characteristics. Figures 12 and 13 illustrate the effect of formed SiC and NbC on the microhardness and compressive strength of the intermetallic alloy during sintering, along with the calculated mean value, variance, and standard deviation of the results obtained, which are tabulated in Tables 4 and 5. As can be seen in the figures shown here, the microhardness and compressive strength of the nanocomposite materials improve significantly as the amount of SiC and NbC present in them increases. The microhardness and compressive strength values of Sample 1 are 144.25 HV and 223.05 MPa, respectively, and were taken as a reference. The highest microhardness and compressive strength values are 244.95 HV and 298.77 MPa, respectively, for Sample 5, which is improved by about 55.69% and 33.95%, respectively, compared to Sample 1. As shown in Figs. 14 and 15, the non-destructive ultrasonic technique was employed to determine the ultrasonic velocities and calculate the elastic moduli for all samples. According to the information presented here, the velocities and elastic moduli of nanocomposites have the same direction as the strength and microhardness of these materials. The velocities and elastic moduli rise considerably when the amount of reinforcement formed is increased. The longitudinal velocity values of Samples 1–5 are 5658.20, 5738.65, 5886.61, 6168.36, and 6701.03 m/s, respectively, and the shear velocity values for the same samples are 3215.60, 3260.49, 3335.26, 3465.19, and 3710.16 m/s, respectively. Additionally, as an example of the elasticity group, the Young’s modulus for Sample 1 is 180.26 GPa. This value rises to 182.95, 189.06, 199.39, and 219.51 GPa for Samples 2, 3, 4, and 5, respectively. These increases are about 1.49%, 4.88%, and 21.77%. The mechanical behavior of the intermetallic alloy is significantly improved when activated carbon is added to the Fe–Si–Nb alloy system, as it promotes the in-situ production of both SiC and NbC. SiC is finely distributed throughout the intermetallic alloy matrix and functions as an efficient barrier to dislocation motion, inhibiting permanent deformation. It is distinguished by its remarkable hardness, high Young’s modulus, and strong covalent bonding^41,50,51^. The alloy’s microhardness, compressive strength, and elastic moduli are all improved by this dislocation blockage. Furthermore, during solidification, SiC particles encourage heterogeneous nucleation, which significantly improves grain refinement. Smaller grains naturally enhance strength through the Hall–Petch process, resulting in a microstructure that is stronger and more stable^52,53^. Meanwhile, another potent strengthening mechanism is introduced by the synthesis of NbC. Because of their high hardness, poor solubility, and thermal durability, NbC precipitates occasionally as nanoscale particles throughout the matrix or at grain boundaries. When exposed to heat, these particles effectively pin the borders and prevent grain coarsening. Previous mechanical qualities are further enhanced by this pinning action, which maintains the structure’s homogeneity and fine grain^48^. When SiC and NbC are present together, their strengthening effects become synergistic rather than additive; dispersion strengthening creates a multi-mechanism reinforcing effect in conjunction with precipitation hardening and grain refining. Furthermore, the inclusion of both carbides significantly improves the composite’s overall stiffness and load-bearing capability, as their moduli are substantially larger than those of the iron matrix. As a result, the Fe–Si–Nb alloy is transformed into a significantly harder, stiffer, and structurally superior material with improved structural stability through the addition of activated carbon, which facilitates the production of SiC and NbC. This makes the alloy ideal for demanding engineering applications. These findings are in excellent agreement with those mentioned in Refs^54–57^.

**Table 4:** The mean value, variance, and standard deviation of microhardness value of all samples.

Sample	Mean value	Variance	Standard deviation
Sample 1	144.25	0.272	0.5215
Sample 2	156.22	0.416	0.6450
Sample 3	175.26	0.292	0.5404
Sample 4	200.35	0.417	0.6455
Sample 5	244.95	0.556	0.7457

**Table 5:** The mean value, variance, and standard deviation of compressive strength value of all samples.

Sample	Mean value	Variance	Standard deviation
Sample 1	223.05	0.436	0.6603
Sample 2	231.36	0.676	0.8222
Sample 3	243.39	0.556	0.7457
Sample 4	264.27	0.54	0.7348
Sample 5	298.77	1.096	1.0469

### Magnetic properties

**Fig. 16:**
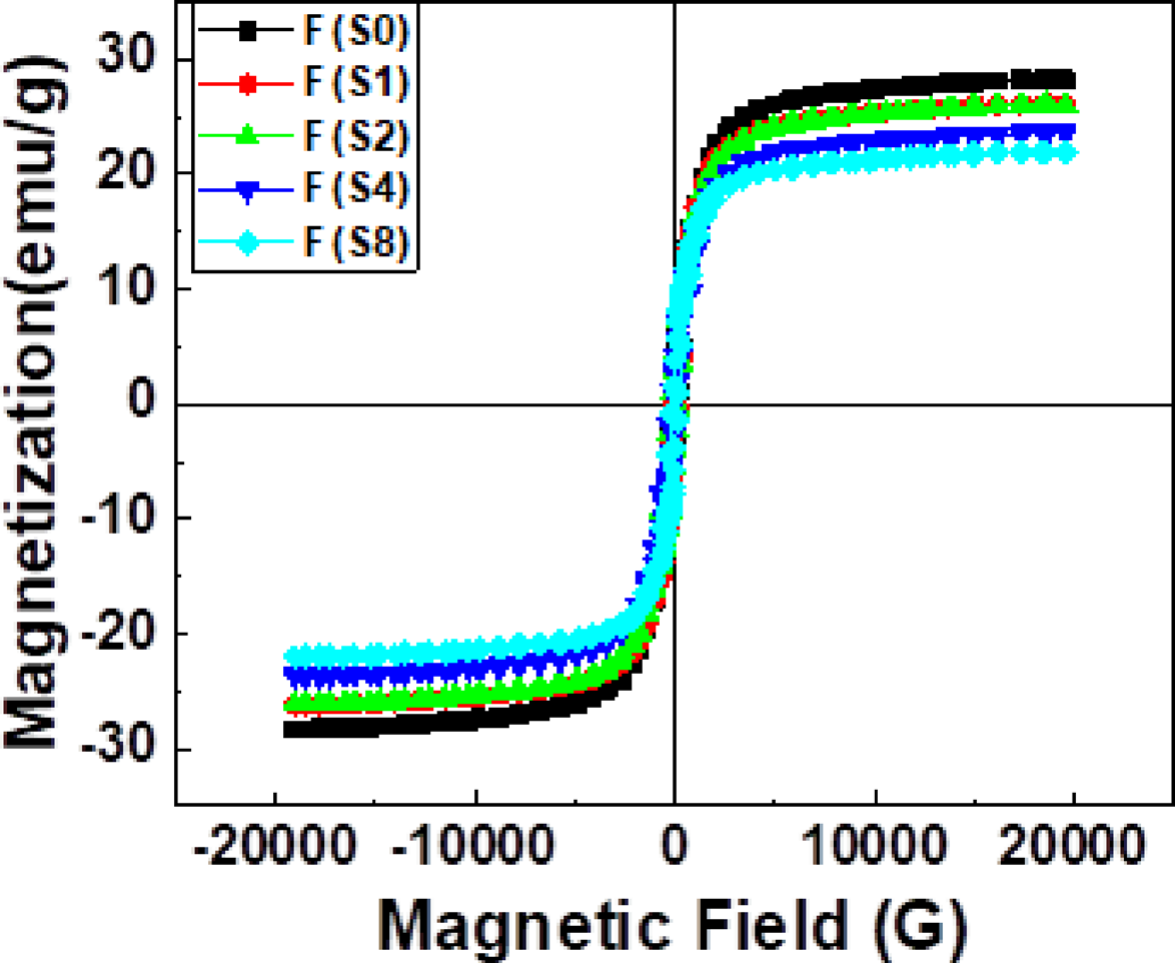
Magnetic hysteresis loops of all the samples.

VSM was used to measure the magnetic field and strength of the samples. While the key magnetic parameters, such as saturation magnetization (M_s_), remanent magnetization (M_r_), coercivity (H_c_), and squareness (M_r_/M_s_), are summarized in Table 6, the magnetic hysteresis (M–H) curves for samples with various activated carbon ratios are shown in Fig. 16. A typical ferromagnetic M-H loop was seen in the samples. It is evident from the hysteresis loops that these samples exhibit soft magnetic properties. The samples’ magnetic behavior is influenced by the carbon content, as seen by notable patterns in the magnetic properties. From 168.06 G for sample 1 to 132.9 G for Sample 5, the coercivity steadily decreases as the concentration of activated carbon rises, indicating that higher activated carbon content reduces resistance to magnetization changes. This decrease in coercivity suggests that carbon doping likely modifies the microstructure, which could reduce internal stresses or defects that pin the boundaries of magnetic domains and facilitate magnetization reversal^58^. Similarly, the saturation magnetization decreases from 28.313 emu/g for Sample 1 to 21.975 emu/g for Sample 5 as the amount of activated carbon increases. This drop is caused by the non-magnetic carbon particles in the alloy diluting the overall magnetic moment per gram of material^59^. Moreover, activated carbon may alter the environment around iron atoms, reducing the number of connections available for magnetic exchange and, consequently, the amount of magnetism. There is some variability but no obvious trend in the retentivity data, which vary from roughly 3.128 to 3.985 emu/g. This suggests that intrinsic material properties and magnetic domain stability may dominate residual magnetism after the external field is removed, making it less vulnerable to carbon doping^60^. With a range of 0.1322 to 0.1758, the squareness ratio fluctuates a little but does not exhibit a consistent trend. This parameter displays the form of the hysteresis loop, the degree of magnetic anisotropy, and domain alignment. The observations suggest a complex relationship, possibly due to the contradictory effects on magnetic interactions and domain wall motion, even though carbon may have an impact on microstructural anisotropy. Anisotropic constant (K) values were computed using the following relation^61^:

**Table 6:** Magnetic parameters for all samples: coercivity, magnetization, retentivity, squareness, and anisotropic constant.

Sample name	Coercivity (G)	Magnetization (M_s_) (emu/g)	Retentivity (M_*r*_) (emu/g)	Squareness SQ (M_*r*_/ M_s_)	Anisotropic constant (K)
Sample 1	168.06	28.313	3.845	0.1358	4855.39059
Sample 2	167.5	26.203	3.985	0.1521	4478.57398
Sample 3	165.1	26.08	3.641	0.1396	4393.68163
Sample 4	157.4	23.652	3.128	0.1322	3798.80082
Sample 5	132.9	21.975	3.863	0.1758	2980.07908

Finally, the anisotropy constant falls from 4855.39 for Sample 1 to 2980.08 for Sample 5. This drop indicates that activated carbon doping reduces the magnetic anisotropy energy barrier, enabling magnetic moments to be reoriented in the presence of an external field. Lower anisotropy constants may improve the soft magnetic behavior of these materials, which would be beneficial for applications requiring low energy losses. To determine the type of exchanges that exist or do not, the squareness ratio, SQ = (Mr/Ms), is listed in the table. Our analysis indicates that the samples’ SQ value is less than 0.5, indicating that exchange coupling is the mechanism by which the particles interact magnetostatically. Reinforcing the intermetallic base with activated carbon consistently reduces coercivity, magnetization, and the anisotropy constant; retentivity and squareness exhibit less consistent changes. By altering the alloy’s microstructural features and magnetic domain dynamics, these patterns show how controlled carbon doping can be utilized to tailor magnetic properties.

## Conclusion

This article focuses on using a carbothermal reaction during powder metallurgy sintering technique to reuse Fe waste and produce Fe_3_Si intermetallic-based nanocomposites reinforced with in-situ carbide ceramics (SiC and NbC). The study concluded with the following findings:

- This approach to recycling Fe waste offers a more sustainable solution and mitigates the extra expenses associated with waste disposal.
- Following the milling process, it is essential to note that the incorporation of varying percentages of activated carbon particles significantly enhanced the grain refinement of the Fe-Si-Nb alloy and reduced the particle size of the composite powders to an ultrafine dimension of approximately 45.7 nm.
- The HRSEM microstructure appears to show a high degree of deification in the intermetallic alloy, which decreases with the addition of activated carbon.
- The bulk and relative densities of the intermetallic base progressively decreased with an increased amount of in-situ reinforcement, changing from 6.91 to 6.23 g/cm³ and from 97.68% to 93.27%, respectively. In contrast, porosity increased from 2.32% to 6.73%.
- The CTE values of the prepared samples experienced a reduction, decreasing from 10.21 × 10^− 6^ / °C to 8.50 × 10^− 6^ /C. This reduction is indicative of the favorable impact that in-situ ceramic reinforcements have on enhancing the thermal stability of the prepared samples.
- Remarkable improvement of the mechanical properties of the intermetallic base, including microhardness, strength, and modulus of elasticity, after forming various amounts of reinforcements. For example, the microhardness values of Samples 1 to 5 are 144.25, 156.22, 175.26, 200.35, and 244.95 HV, respectively.
- In-situ ceramics have a negative impact on the magnetic properties of the intermetallic base. Still, this reduction is not significant and therefore does not have a negative effect when these composites are used in applications.

## Data Availability

The datasets used and/or analysed during the current study available from the corresponding author on reasonable request.
